# Long-term effectiveness of a gambling intervention program among children in central Illinois

**DOI:** 10.1371/journal.pone.0212087

**Published:** 2019-02-11

**Authors:** Jinma Ren, Kirk Moberg, Heidi Scuffham, Dongming Guan, Carl V. Asche

**Affiliations:** 1 Center for Outcomes Research, Department of Medicine, University of Illinois College of Medicine at Peoria, Peoria, IL, United States of America; 2 UnityPoint Health Illinois Institute for Addiction Recovery, Peoria, IL, United States of America; 3 Center for Pharmacoepidemiology and Pharmacoeconomic Research, University of Illinois at Chicago College of Pharmacy, Chicago, IL, United States of America; Universitat de Barcelona, SPAIN

## Abstract

Youth gambling is an increasing concern. As a response, the “Don’t Gamble Away our Future (DGAOF)” program has been implemented among children in central Illinois. We aim to assess the long-term effectiveness of this school-based youth gambling prevention program in Illinois using the data from 2005 to 2009. The intervention included interactive PowerPoint presentations and prevention materials in parent packets. Students aged 8 to 18 years were eligible to participate in the intervention and the questionnaire pre-post knowledge tests (total score 0–9). Students in 5th grade and above also received a gambling behavior screen test using the Modified South Oaks Gambling Screening for Teens (MSOGST) for identifying probable gamblers. Multivariable generalized mixed models were conducted to detect the effects of a 5-year youth gambling prevention program as controlling potential confounders. A total of 16,262 and 16,421 students completed pre-post tests and MSOGST tests, respectively. Of 16,262, half were female, the majority (76.1%) were from senior high school, and 21.3% received the intervention at least twice. The median gap between interventions was 368 days. Students receiving multiple interventions had higher scores on the pre-test as compared to those receiving a single intervention (P<0.001 for all comparisons among groups), and they demonstrated an increasing trend of awareness about gambling over time (P<0.001 for multiple interventions; P = 0.538 for single intervention). The prevalence of problem gambling had decreased among students receiving the intervention twice as compared to receiving the intervention once (7.9% versus 9.4%; OR = 0.89, 95% CL: 0.82–0.97). However, this effect was not confirmed among students receiving the intervention three or more times. In conclusion, the DGAOF program has demonstrated a positive long-term impact on increasing gambling knowledge and partially reducing pathological gamblers through direct training. It suggests that multiple repeated interventions are important for youth gambling prevention.

## Introduction

Youth gambling has been the subject of increasing concern in the United States and throughout the world.[1] The current generation of youth has grown up in an era where gambling opportunities are widespread.[2,3] New forms of gambling via the Internet, mobile phone and interactive television have been attracting more young gamblers.[4] Social casino games may facilitate the transition to online gambling among younger teenagers (i.e., 12–14 years old), as a result of the ease of accessibility and early exposure.[5] Sixty-eight percent of young Americans have gambled in 2007, 11% gambled twice per week or more, 6.5% were at-risk or pathological gamblers, and 2.1% were pathological gamblers.[6] A study in central Illinois indicated that 10% of youth were “probable pathological gamblers”.[7]

Gambling that begins in adolescence may be associated with elevated severity of problems throughout the life span of older adult pathological gamblers.[8] Pathological gamblers are more likely to use tobacco, alcohol, and other illicit drugs.[9,10] Previous studies have indicated that gambling is significantly associated with criminal activity.[11,12] Thus, there is a critical need to develop and implement effective intervention programs to reduce gambling in youth. Youth gambling prevention has been rapidly developed in the past few years on the basis of some widely recognized theoretical models.[13–15] One of the broadly accepted conceptualizations of youth problem gambling and consequently youth gambling prevention has been the Dickson’s model.[16] In this model, pathological gambling is listed as one of adolescent risk behaviors, and a wide range of factors work together to influence whether an adolescent will engage in gambling behavior including being male (biology), access to gambling venues (social environment), models for deviant behavior (perceived environment), depression and anxiety (personality), and poor coping skills (behavior). Grounded on this model, school-based youth gambling prevention programs have been implemented including both abstinence and harm reduction elements.[17–19]. These programs usually involves lectures, small group discussions, interactive games and exercises, and questionnaire surveys. In addition, it has been recognized that parents should be taken into account in the development of youth gambling prevention programs. A survey in Macau showed that half of parents did not approve underage gambling but 81% taught their underage children to play different gambling games.[20]

Despite different approaches (e.g. classroom-based or media education), school-based youth gambling prevention programs have demonstrated their short-term effects, such as improving gambling knowledge and changing attitudes towards gambling after intervention.[17–19] However, the impact on actual gambling behavior has not been well-established as there are only few studies that have assessed the long-term effectiveness of youth gambling interventions in behavioral change in the literature.[21] Previous studies have demonstrated a promise effect on the gambling behavioral change[22,23], except one study reported no significance influence on lifetime gambling in a short-term program.[18] It suggests that studies with longer follow-up period are needed to test the long-term effect of such an intervention.

As a response, the Illinois Institute for Addiction Recovery (IIAR) has implemented a gambling awareness prevention program “Don’t Gamble Away our Future” (DGAOF, also called intervention in this paper) and collected data for the purpose of evaluation since 2005.[7] An analysis of DGAOF program data after one year found that this program was successful in increasing knowledge of gambling.[7] However, the long-term effects have not been evaluated in relation to knowledge of gambling and reductions in problem gambling. In this study, we examine whether the DGAOF program, in delivering multiple interventions over time, decreases the prevalence of problem gambling by increasing gambling knowledge among youth. We also explore the effects of peer-education by examining if the DGAOF program increases gambling knowledge among untrained youth over time.

## Materials and methods

This study has been approved by the Peoria Institute Review Board located in the University of Illinois College of Medicine at Peoria. Although it was an educational program, consent was still obtained from school principals first, and the schools informed participants’ parents in writing through parent folder.

### Study design

This is an intervention study of young students participating in the DGAOF program in central Illinois, which was initiated by the IIAR in 2004 (data collection started in 2005) and conducted annually thereafter. This study only analyzed the data between 2005 and 2009 because the IIAR has not been ready to release the later data. Students initially enrolled in the DGAOF program were assessed at baseline, and most of them were also assessed in subsequent years when additional new students were recruited. We examined the long-term effect of DGAOF program through: 1) observing change in gambling knowledge over time among students with multiple participations; 2) observing change in gambling knowledge from 2005 to 2009 among new participants (effects of peer-education); and 3) determining if multiple annual interventions increase gambling knowledge and decrease prevalence of problem gambling among students compared to participants with only one intervention.

### Intervention

Interactive PowerPoint presentations with students were conducted to introduce in-depth prevention material for each age group (S1 Appendix, S2 Appendix, and S3 Appendix). For instance, probability concept was not introduced for students in primary schools. Presentations were held in classrooms with approximate 30 students learning at once. Each presentation lasted 45–60 minutes including interactive discussions and games. An average of 14 presentations were provided for each school annually in order to cover all eligible students. The presentation was usually held in Health classes in high schools, in Physical Exercise classes in middle schools, and in each individual classroom in primary schools. At the conclusion of every presentation, each student was given a parent packet to take home, which included a Gambling Fact Sheet (S4 Appendix) and a Parent Letter (S5 Appendix) that explained the DGAOF program in details, a wrist bracelet with the *Don’t Gamble Away Our Future* logo on it, and an interactive CD-ROM that provided education about problem gambling in an engaging format.

### Data collection and measurement

A pre- and post-knowledge test was self-developed to determine the knowledge gained as a result of the prevention materials. The post-test was conducted immediately at the end of session. The test included 14 questions for high school students (S6 Appendix), 12 questions for junior high school students after removing items 6 and 7, and 9 questions for primary school students after removing items 5–8 and 11 because those questions are not relevant to that age group. Here is an example of items “*The definition of gambling is*: *betting money on something when the outcome is uncertain*” as well as its response options “*True/False*”. For each student, his/her total knowledge score was multiplied by 9 and divided by the number of questions in order to make an average score within a range of 0 to 9. Higher scores are indicative of greater knowledge.

Students at 5^th^ grade and older were also assessed (post-test only) for their current gambling behavior through the Modified South Oaks Gambling Screen for Teens (MSOGST) as shown in S7 Appendix. The MSOGST is one of the best tools for evaluating adolescent gambling problems despite the questions raised regarding the validity.[24] It includes 16 questions, but questions 1, 2, 3 and 12 are not counted for scoring. Most of the items are “yes/no” questions, such as “*Did you ever gamble more than you intended to*?” The score range is from 0 to 21 because question 16 comprises 10 sub-questions. Higher scores indicates higher likelihood of pathological gambler.

In addition, school name, date of presentation, student names, gender, grade, age, and their unique identifier numbers were collected. All de-identified supplement data were online (S1 Dataset and S2 Dataset).

### Procedure

For the purpose of enrollment, we tried to reach out to all schools and detention centers in Midwestern Illinois. We actively contacted the principals or administrators of 90 schools that we had known or enrolled via mass media advertising (e.g. television). Eventually, there were 12 primary schools, 29 junior high schools, 24 high schools, 1 detention center and 1 youth prison participating in this program during the study period. Forty-eight percent of enrolled schools participated in this program multiple times. The enrolled schools were distributed in nine counties including Peoria, Tazewell, Woodford, Fulton, Sangamon, McLean, Henry, Morgan, and Stark. In each enrolled school, all students from grade 3 to 12 (8–18 years old) were invited to receive our intervention training.

Eligible students were only invited to attend the DGAOF program once per calendar year, but they were allowed to participate in this program multiple times over the years. For each intervention, participants were asked to complete a pre- and post-knowledge test and/or a MSOGST test (post-test only) in the classroom at the same day of presentation. The completed questionnaires were stored in a locked office, and entered into a secured computer at the IIAR. Only de-identified data were shared with our team members in the University of Illinois College of Medicine at Peoria for analysis.

### Sample size

At the phase of study design, a power analysis was conducted based upon two outcomes: knowledge score and prevalence of problem gambling. The previous analysis showed the pre- and post-test scores were 6.11 (standard deviation, SD = 2.09) and 7.43 (SD = 1.95), respectively, and the prevalence of problem gambling was 10%.[7] Thus, in our sample size calculation, we presumed that 1) the knowledge score would increase 1.32 (6.11 versus 7.43) after multiple interventions; 2) the prevalence of problem gambling would decrease 2 percentage points at 1-year post-intervention (10% versus 8%); and 3) approximately 20% of initial participants would receive at least two interventions. Given a significance level of 0.05 and a statistical power of 90%, we estimated that a minimum sample size of 180 and 15,678 are needed to examine the changes of knowledge score and problem gambling prevalence, respectively. Because the change of prevalence (10% versus 8%) was chosen arbitrarily, we also did a post-hoc power analysis, which demonstrated that our study had a statistical power of 87.2% to determine the impact of intervention on prevalence change (9.4% versus 7.9%) at 1-year post-intervention. Therefore, the sample size (more than 16,000) in this study was sufficient to examine our hypotheses.

### Statistical analysis

Analyses were done with SAS 9.4 (SAS Institute Inc., Cary, NC, USA). In order to describe the baseline characteristics and outcomes, frequency distributions were reported for categorical variables, and mean and standard deviation values were calculated for continuous variables. Less than 3% of BMI data were missing, which were replaced by mean values in analysis. A statistical significance level of 0.05 was set for all relevant tests in this study.

In order to analyze whether the intervention increases gambling knowledge in both short- and long-term period, we used a generalized linear mixed model to examine the change of knowledge score between pre- and post-interventions, and compare the difference scores among students receiving intervention once, twice, and three or more times. It should be noted that participants who only competed the program on one occasion were assessed at multiple follow-up time-points. Further, in our sub-analysis, we examine the trend of knowledge score over time among students receiving single and multiple interventions using the pre-test scores only (score before intervention each year). The trend of knowledge score over time for those new participants could reflect the effect of DGAOF program by peer-education. The multivariable analyses were adjusted by school, grade, gender, and year in order to control confounders. Year was set as both categorical and continuous variables for estimating the effect each year and the trend over time, respectively.

The score of MSOGST was categorized into three levels: 5 or greater for probable pathological gambler, 1–5 for some problem with gambling, and 0 for no gambling problem.[7] The association between the number of program participations and the prevalence of problem gambling was examined in a cumulative logistic regression model that included other fixed-effect variables of school, grade, gender, and year as well as a random effect of students. Odds ratio (OR) and 95% confidence limits (95% CL) were reported to measure the strength of association.

## Results

### Participant characteristics

Our final analysis included 16,262 students who finished pre/post-tests of gambling knowledge and 16,421 students who took MSOGST tests for screening pathological gamblers. The number of students in this program varied each year during the study period. Of these, the majority (76.1%) were high school students, approximately half were female, and 21.3% received the intervention twice or more. The median gap between interventions was 368 days with an interquartile range of 245 to 543 days. Some high school students were not able to receive multiple interventions after graduation (Table 1).

**Table 1:** Demographics of students in the analysis. MSOGST, Modified South Oaks Gambling Screen for Teens.

Variables	Students receiving a pre- and post-test				Students receiving a MSOGST test			
	Single intervention		Multiple interventions		Single intervention		Multiple interventions	
	Number	%	Number	%	Number	%	Number	%
**Year**								
**2005**	1212	9.5	274	7.9	1081	9.2	348	7.5
**2006**	3434	26.9	444	12.8	3676	31.2	1275	27.4
**2007**	3860	30.2	1319	38.0	3398	28.9	1305	28.0
**2008**	2640	20.6	905	26.1	2159	18.4	992	21.3
**2009**	1644	12.9	530	15.3	1453	12.4	734	15.8
**School**								
**Detention center**	194	1.5	74	2.1	174	1.5	73	1.6
**High school**	10451	81.7	1919	55.3	8993	76.4	2495	53.6
**Junior high**	1093	8.6	1134	32.7	2600	22.1	2086	44.8
**Primary school**	1052	8.2	345	9.9	0	0.0	0	0.0
**Gender**								
**Male**	6495	50.8	1716	49.4	6019	51.2	2267	48.7
**Female**	6295	49.2	1756	50.6	5748	48.8	2384	51.2
**Grade**								
**3**	570	4.5	131	3.8	0	0.0	0	0.0
**4**	491	3.8	215	6.2	0	0.0	0	0.0
**5**	312	2.4	247	7.1	707	6.0	240	5.2
**6**	287	2.2	245	7.1	737	6.3	472	10.1
**7**	294	2.3	327	9.4	592	5.0	664	14.3
**8**	275	2.2	341	9.8	630	5.4	738	15.9
**9**	6707	52.4	1101	31.7	5871	49.9	1502	32.3
**10**	2070	16.2	620	17.9	1748	14.9	759	16.3
**11**	972	7.6	147	4.2	786	6.7	179	3.9
**12**	812	6.4	98	2.8	696	5.9	100	2.2

### Gambling knowledge

**Fig 1:**
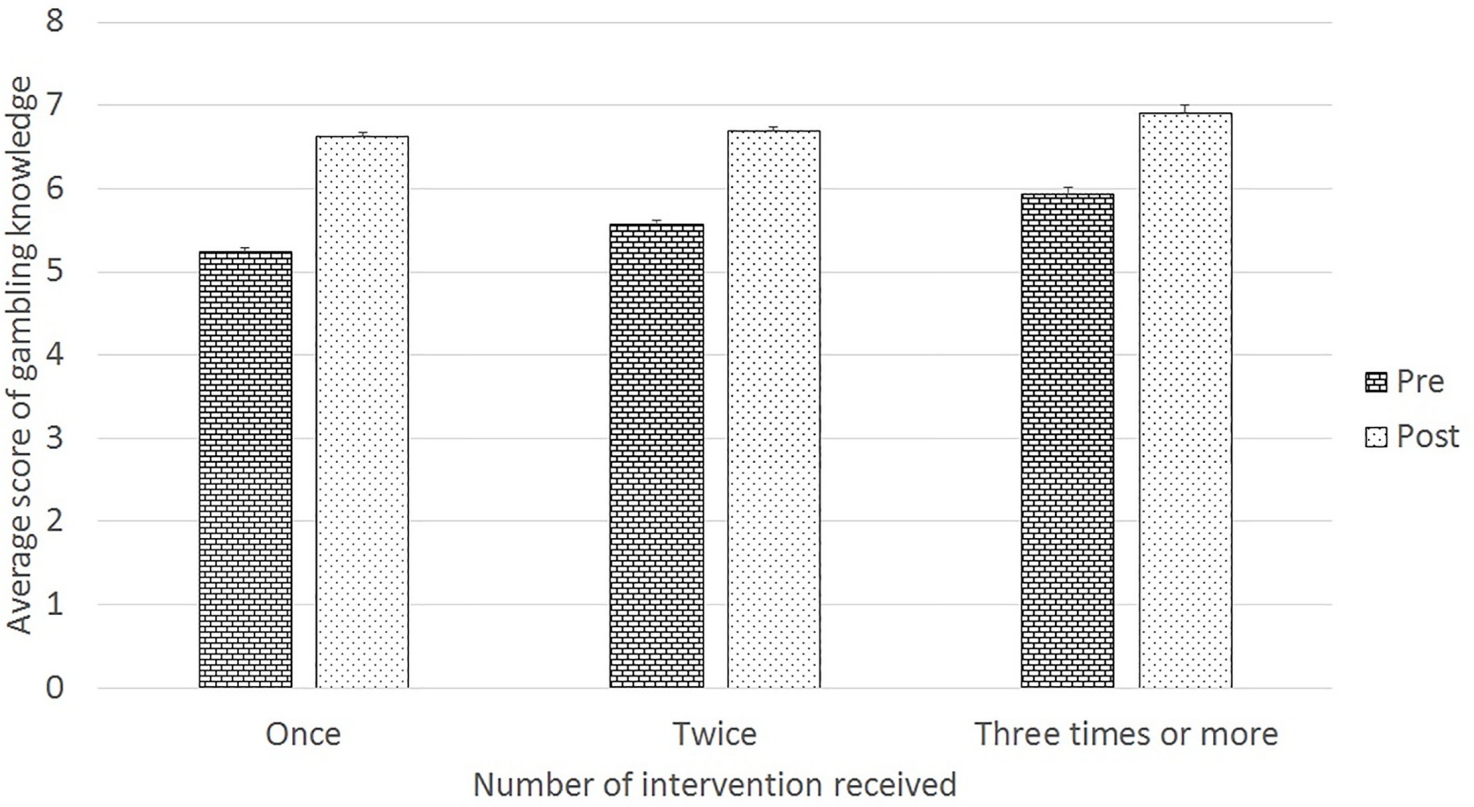
Pre- and post-test scores of gambling knowledge among students receiving single or multiple interventions. * A generalized linear mixed model was used to examine the change of knowledge score between pre- and post-interventions, and compare the scores among students receiving once, twice, and 3+ interventions. P<0.001 for overall pre- vs post-tests; for pre-test only, P<0.001 for all comparisons among the tree groups of intervention once, twice, and 3+; for post-test only, P values were 0.007, <0.001, and 0.128 for intervention twice vs. once, 3+ vs. once, and 3+ vs. twice, respectively. The bars and upper caps stand for means and upper limits of confidence interval.

After controlling for school, grade, gender and year, our results demonstrated that students receiving multiple interventions had higher scores of pre-tests as compared to those receiving a single intervention (average difference from 0.3 to 0.7, P<0.001 for all comparisons among the tree groups of intervention once, twice, and three or more times, Fig 1). This association was also observed for post-tests (average difference from 0.1 to 0.3, P values were 0.007, <0.001, and 0.128 for intervention twice vs. once, three or more times vs. once, and three or more times vs. twice, respectively).

**Fig 2:**
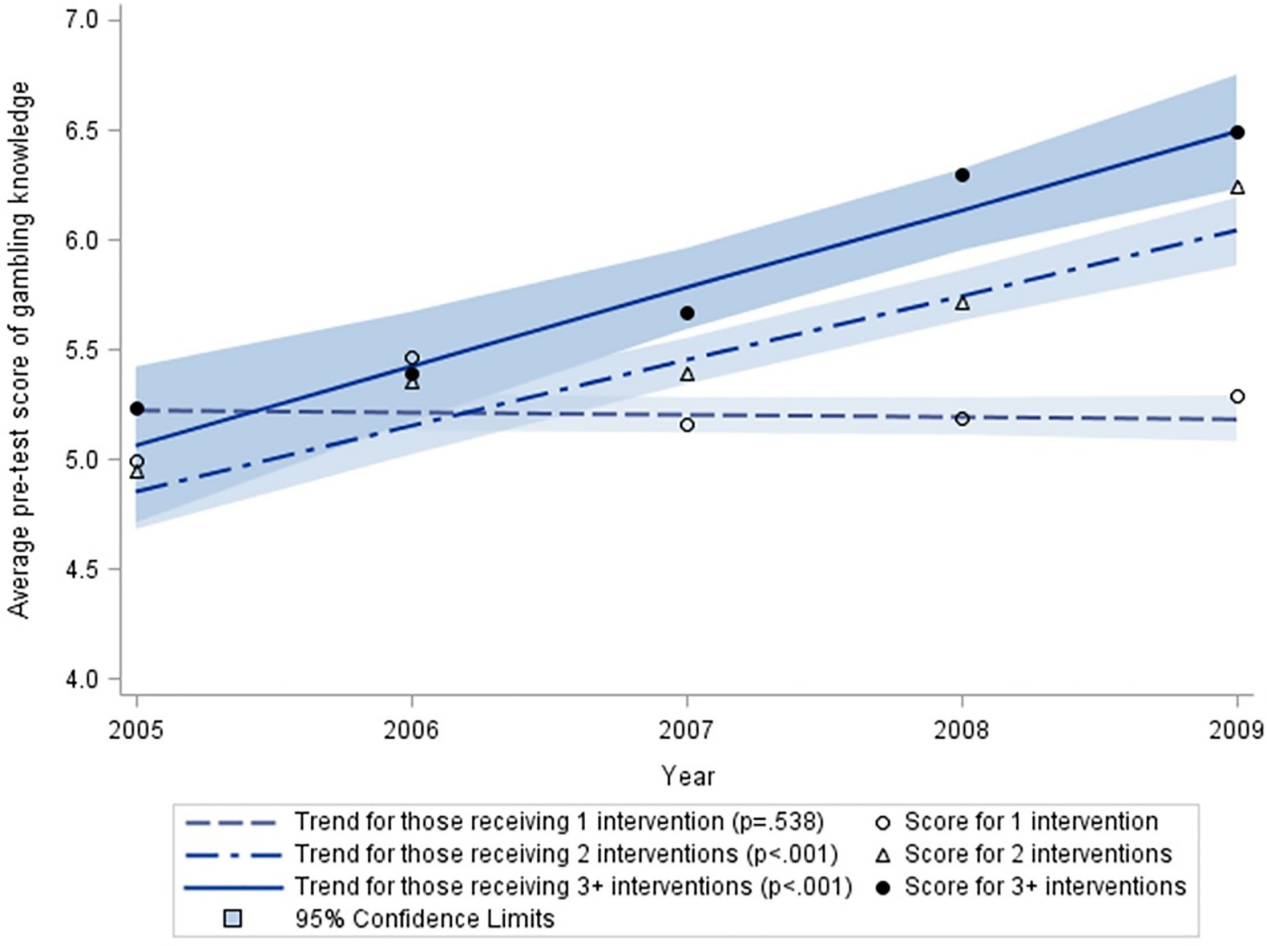
Trend of pre-test score of gambling knowledge among students receiving single or multiple interventions. * A generalized linear mixed model was used to examine the trend of knowledge score over time among students receiving single and multiple interventions using the pre-test scores only (score before intervention each year). Based on the pre-test data, this analysis demonstrated that the gambling knowledge increased over time (from 2005 to 2009) among students receiving multiple interventions (P<0.001). However, the increased trend of gambling knowledge was not found among students receiving a single intervention only (P = 0.538).

As depicted in Fig 2, the sub-analysis only using pre-test data showed that the gambling knowledge increased approximately 1.5 points of score over time (from 2005 to 2009) among students receiving multiple interventions (P<0.001). However, the increased trend of gambling knowledge was not found among students receiving a single intervention only (P = 0.538), which indicated that this program had not shown an impact on untrained youth by peer-education.

### Prevalence of problem gambling

The unadjusted prevalence of problem gambling was 9.4%, 7.9%, and 8.8% among students receiving intervention once, twice, and three or more times, respectively. In the multivariable analysis (Table 2), we found that the risk of problem gambling was decreased among students receiving intervention twice compared to once only (OR = 0.89, 95% CL: 0.82–0.97), however, this effect was not confirmed among students receiving intervention three or more times (OR = 1.11, 95% CL: 0.97–1.27). We also found that males were more likely to be classified pathological gamblers than females (OR = 2.60, 95% CL: 2.44–2.78), and students in detention center were more likely to be classified pathological gamblers than those in junior high school (OR = 2.68, 95% CL: 1.78–4.04). Compared to those in junior high school, students in high school did not have a significantly higher risk of problem gambling (OR = 1.36, 95% CL: 0.81–2.30).

**Table 2:** Multivariable analysis of problem gambler prevalence using a mixed-effects cumulative logistic regression model.

Variable	Label	Parameter estimate	Standard error	P value	Odds ratio and 95% confidence limits
**School**	**Junior high**	reference			
	**Detention center**	0.987	0.209	<0.001	2.68 (1.78, 4.04)
** **	**High school**	0.310	0.266	0.244	1.36 (0.81, 2.30)
**Grade**	**5**	reference			
	**6**	0.118	0.095	0.211	1.13 (0.94, 1.35)
** **	**7**	0.253	0.094	0.007	1.29 (1.07, 1.55)
** **	**8**	0.355	0.092	0.000	1.43 (1.19, 1.71)
** **	**9**	-0.076	0.275	0.782	0.93 (0.54, 1.59)
** **	**10**	-0.193	0.275	0.484	0.83 (0.48, 1.41)
** **	**11**	-0.373	0.279	0.181	0.69 (0.40, 1.19)
** **	**12**	-0.361	0.283	0.202	0.70 (0.40, 1.21)
**Gender**	**Female**	reference			
** **	**Male**	0.956	0.033	<0.001	2.60 (2.44, 2.78)
**Year**	**2005**	reference			
** **	**2006**	-0.040	0.063	0.528	0.96 (0.85, 1.09)
** **	**2007**	0.120	0.064	0.061	1.13 (0.99, 1.28)
** **	**2008**	0.090	0.067	0.180	1.10 (0.96, 1.25)
** **	**2009**	-0.131	0.072	0.069	0.88 (0.76, 1.01)
**Number of interventions**	**1**	reference			
	**2**	-0.116	0.041	0.005	0.89 (0.82, 0.97)
	**3**	0.103	0.068	0.133	1.11 (0.97, 1.27)

## Discussion

In this study, we utilized a 5-year data to evaluate the long-term effect of DGAOF program among children and adolescents in central Illinois. We found that the DGAOF program not only increased students’ gambling knowledge after intervention immediately, but also retained the effect for at least one year. The long-term effect of DGAOF program on awareness of gambling and prevalence of problem gambling was further enhanced when students received twice interventions periodically. However, this program has not demonstrated an effect on new students by peer-education in this area.

Youth gambling prevention has demonstrated significant short-term effects, especially on increasing gambling knowledge, just like what the DGAOF did[7]. However, knowledge may be insufficient to induce changes in problem gambling behavior.[21] This leads to prevention efforts focuses on addressing gambling misconceptions. Walther et al. examined the short-term effects of a media education prevention for sixth and seventh grade students on their gambling knowledge, attitudes and behaviors.[18] They found that students in the treatment group had an increase in gambling knowledge, and reduction in the both problematic gambling attitudes and current gambling behavior seven weeks after intervention. Their results also suggested that long-term effects should be taken into consideration when analyzing the effectiveness of youth gambling prevention programs. It usually takes a long time from knowledge increase to behavior change in gambling prevention, and sometimes it is very difficult.[25] Thus, multiple repeated interventions might help children retain awareness about gambling and issues related to gambling, in order to reduce the harm of gambling. The merit of our study is to further provide evidence for a long-term effect of school-based youth gambling prevention programs. In our study, we found that the gambling knowledge significantly increased and the prevalence of problem gambling slightly declined among students receiving multiple interventions rather than a single intervention. These findings suggest that it remains important to continue the DGAOF program in central Illinois in order to reduce the harm of youth gambling.

Sometimes, youth gambling prevention program may be questioned because of inconsistent findings in the effectiveness of providing prevention programs regarding addictive behaviors to adolescents in the literature.[26–28] In fact, the main reason might be because there are comparatively more programs addressing substance abuse than other mental health problems.[29] Gambling addiction is different from smoking and drug addictions although they have some commons. Compared to gambling addiction, both smoking and drug addictions involve not only behaviors but also poisoning chemicals, which is more complicated and increases the difficulty of prevention. Even if the target population are drug users, interactive prevention programs rather than simple education could work well.[27] In addition, the inconsistent findings might also be explained by another reason that very few youth prevention programs have been designed for a long period of time. Thus, the findings of DGAOF program exactly fit the gap and encourage us to continue to implement youth gambling prevention programs.

Adolescent problem gambling shares all health compromising outcomes similar to other youth risk behaviors, such as illicit drug use, delinquency, drink-driving, and tobacco use. These outcomes include physical health issues, various social roles, personal development problems and compromises to typical tasks that prepare adolescents for adulthood such as acquiring motivation and skills to maintain a job. Therefore, Dickson suggests that school-based curriculums should be conceptualized into a wider picture of youth problem and risk-taking behaviors.[16] Integrating a gambling education into school social studies might be another feasible approach to let students engage in the gambling prevention. In addition, parents’ attitudes, knowledge, and behavior toward youth gambling should be considered when designing gambling prevention programs in children.[30] In our DGAOF, each student was given a parent packet to take home, which included a Gambling Fact Sheet and a Parent Letter. We believe that the involvement of parents in the program contributes to help adolescents to increase gambling knowledge, address gambling misconceptions, and eventually change their behaviors.

In this study, we also found that pathological gamblers were more likely to be classified among males and students in detention center. Gambling attitude is one of important determinants for problem gambling behavior. A longitudinal study demonstrated that male adolescents were more likely to develop attitude change towards gambling.[31] It is not surprising that detention centers could have more pathological gamblers than regular schools because gambling increases criminal activities.[11] [12]

A few limitations in our study should be noted. First of all, this longitudinal study did not have a randomized control group because the DGAOF has intended to cover all schools in this area. But, the long-term effect of DGAOF still could be detected in our study design by comparing with the baseline and new participants. Secondly, it would be better if we could show a longer trend using the 13-year data (2005–2017), however, the IIAR could only provide the 5-year data so far. Thirdly, potential pathological gamblers might be more likely to repeatedly participate in this program, which may be the reason why the DGAOF could not significantly decrease the prevalence of problem gambling among students receiving intervention three or more times. Fourthly, a regression to the mean should be considered in the interpretation of repeated-measure data. In this study, we believe that a regression to the mean did not have much influence on our results because our data were not extreme on the first measurement. Finally, this study did not collect data from children’s parents although the intervention provided a parent packet for students to take home.

## Conclusions

The DGAOF program among children in central Illinois has demonstrated a positive long-term impact on increasing the knowledge about gambling and partially reducing the prevalence of pathological gambler through direct training, but its influence among untrained students via peer education is limited so far. Our findings suggest that it remains important to continue the DGAOF program in central Illinois, which also could be inferred that multiple repeated interventions are essential for similar youth gambling prevention programs in other area.

## Supporting information

S1 Appendix PowerPoint presentation for primary school students. (PDF)

S2 Appendix PowerPoint presentation for middle school students. (PDF)

S3 Appendix PowerPoint presentation for high school students. (PDF)

S4 Appendix Gambling fact sheets for parents. (PDF)

S5 Appendix A parent letter. (PDF)

S6 Appendix Gambling knowledge questionnaire for high school students. (PDF)

S7 Appendix Modified south oaks gambling screen for teens (MSOGST). (PDF)

S1 Dataset De-identified data for pre and post tests. (SAS7BDAT)

S2 Dataset De-identified data for MSOGST tests. (SAS7BDAT)
