# Supplementary material for: Long-term effectiveness of a gambling intervention program among children in central Illinois
Source: PLoS One. 2019 Feb 11;14(2):e0212087. doi: 10.1371/journal.pone.0212087 (PMC6370280; doi:10.1371/journal.pone.0212087)

Don't GAMBLE AWAY Our Future[SM]

Introductions

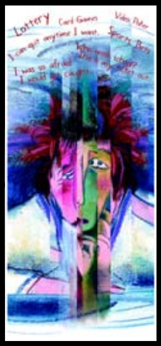

- Brief statement of the goals of the program
- Pre-test

Information about Gambling

Did you know

The effects of a gambling problem can be just as serious as a drug or alcohol problem?

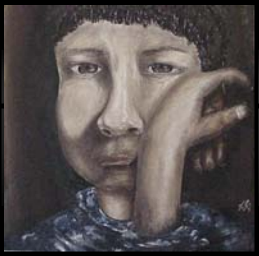

Common Gambling Activities

- Sports betting
- Poker & Blackjack
- Video games
- Flipping cards
- Dice games
- Casino gambling
- Horse racing
- Bingo
- Lottery
- Internet betting
- Scratch tickets
- Video lottery machines
- Betting on games of personal skill

Is it gambling?

- Is betting on a football game for money gambling?

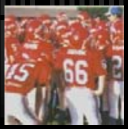

- ANSWER: Yes
- It's gambling because you are risking something of value.

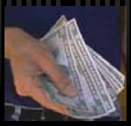

Is it gambling?

- While playing a video game with your friends, everyone agrees to give their favorite game controller to whomever reaches the highest level in the game first.
- Is this gambling?

- ANSWER: Yes
- It is gambling because the winner receives something of value from the other players.

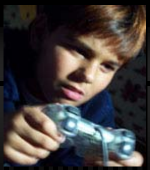

Is it gambling?

- Is it gambling to say to your friends, “I can make more free throws than you.”?

- ANSWER: No.
- Competition is in most cases healthy. There are times, however, when competition can lead to risky gambling related behaviors.

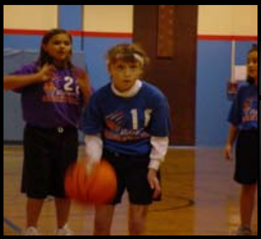

Gambling is...

Risking something of value, when the outcome is uncertain.

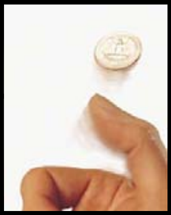

Beliefs About Gambling

- True or False: It is easy to win back money you’ve lost gambling, you just have to gamble long enough for it to happen.

- ANSWER: False
- That is called “chasing the loss” and it only results in losing more money. Rarely do people win back the money they’ve lost gambling.

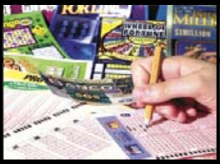

Are the Following Games of Skill or Games of Chance?

- Chess
- Baseball
- Bowling
- Basketball
- Pool
- Golf

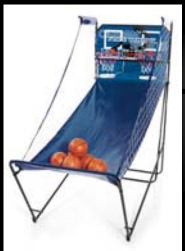

- ANSWER: GAMES OF SKILL
- These are all games that you can improve in, if you practice them.

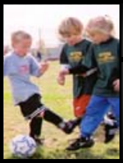

Are the Following Games of Skill or Games of Chance?

- Lotteries
- Slot Machines
- Bingo
- Video Slot Machines
- Roulette
- Craps
- Keno

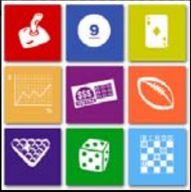

- ANSWER: GAMES OF CHANCE
- These games are all based on chance. Chance is uncontrollable. No one can do anything to improve their performance while playing these games.

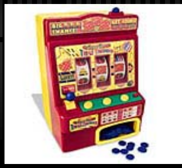

Games of Skill vs Games of Chance

- To be a smart gambler you should
  - a. Practice daily
  - b. Develop a strategy
  - c. Be careful, set limits and stick to them

- ANSWER: C = Be careful, set limits and stick to them.
- Gambling is based on chance. Smart gamblers wait until they are of legal age to gamble, then set betting limits and STOP when they've reached them.

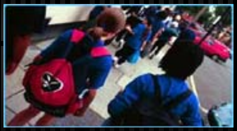

Games of Skill vs Games of Chance

- True or False: To increase your chance of winning while gambling, just follow your gut instincts.

- ANSWER: False
- Chance is uncontrollable. Your gut instincts cannot predict the outcome.

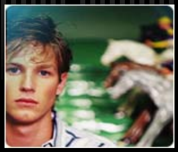

Which answer is a True Statement?

- Gambling every week will result in:
  - a. Improving your gambling skills
  - b. Losing money
  - c. Becoming smarter in Math

- ANSWER: B = Losing money
- Over the long run people will always lose money gambling.

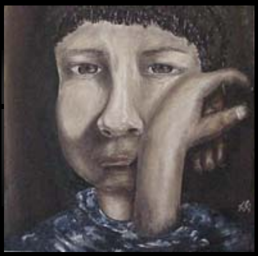

True or False Statements...

- Is this True or False?
  - The more I tell my friends how much money I'm betting, the more I will impress them.

- ANSWER: False
  - True friends will not be impressed by gambling.

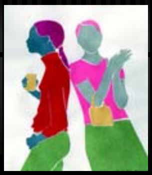

True or False Statements...

- True or False?:
  - To become hooked on gambling, you have to be an adult who has spent many years at it.

- ANSWER: False
  - Young people can become problem gamblers very quickly.

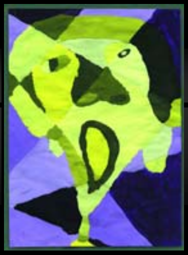

Which answer is a True Statement?

- Gambling is ok if:
  - a. You have a perfect strategy to win
  - b. You need to make some fast money
  - c. You can afford to lose whatever you bet

- ANSWER: C = You can afford to lose whatever you bet.
- There is no strategy to win and you can't get rich quick by gambling.
  - Plan how much you can afford to lose and then stop at that amount.

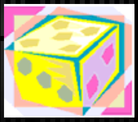

Problem Gambling – Signs & Symptoms

- Changes in behavior
- Changes in mood
- Unexplained need for money
- Carrying dice, cards, lottery tickets, etc.
- Unusual time spent watching sports on TV
- Gambling language in his/her vocabulary
- Late night phone calls from strangers
- Several calls to sports phone on telephone bill
- Having extra spending money

Progression

- Some wins
- Thinking about gambling when not gambling
- Needing to gamble more often and/or with more money
- Losing money and "chasing losses"
- Trying to stop gambling but not being able to do so
- Being irritable when not gambling
- Lying or committing crimes to support gambling
- Feeling desperate
- Losing everything, including hope

- Living life full of hope and enjoyment
- Thinking about school, family, work and the future
- Focusing on taking life one day at a time
- Being wiling to examine self and change things that need changing
- Developing productive coping skills and abilities
- Becoming honest with family and friends
- Actively seeking support and treatment
- Wanting life to be different

More information coming your way...

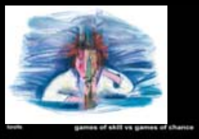

A CD ROM

If You or Someone You Love...

has a problem with gambling or any other substance or behavior, there is help available 24 hours a day

The Illinois Institute for Addiction Recovery

1/800/522-3784

In Conclusion

- Post-test

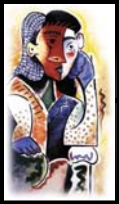

Supplement: S1 Appendix — (PDF) [file pone.0212087.s001.pdf]
