# Supplementary material for: Long-term effectiveness of a gambling intervention program among children in central Illinois
Source: PLoS One. 2019 Feb 11;14(2):e0212087. doi: 10.1371/journal.pone.0212087 (PMC6370280; doi:10.1371/journal.pone.0212087)
Supplement: S2 Appendix — (PDF) [file pone.0212087.s002.pdf]

Don't GAMBLE AWAY Our Future

Introductions

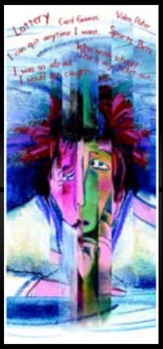

- Brief statement of the goals of the program
- South Oaks Gambling Screen
- Pre-test

Teen vs Adult Gambling

Studies have shown that approximately 80% of teens have gambled in the past year as opposed to 68% of adults who have gambled during the same time.

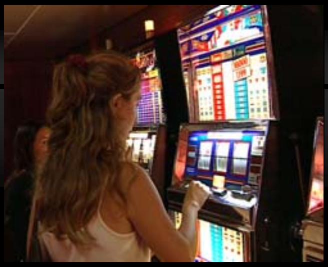

Teen vs Adult Gambling

Did you know.................

The effects of a gambling problem can be just as serious as a drug or alcohol problem?

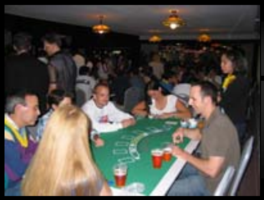

Common Gambling Activities

- Sports betting
- Poker & Blackjack
- Video games
- Flipping cards
- Dice games
- Casino gambling
- Horse racing
- Bingo
- Lottery
- Internet betting
- Scratch tickets
- Video lottery machines
- Betting on games of personal skill

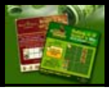

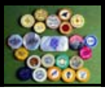

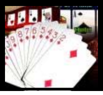

Is it gambling?

- You participate in a March Madness basketball pool with people from your school.
- Is this gambling?

- ANSWER: Yes
  - It is gambling because the winner receives money from the other participants.

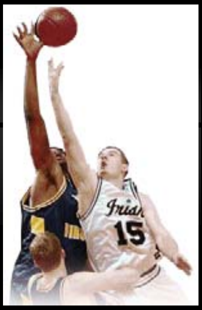

Is it gambling?

- Is betting on a high school football game for money gambling?

- ANSWER: Yes
  - It's gambling because you are risking something of value.

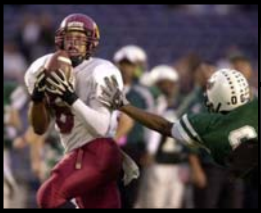

Is it gambling?

- While playing a video game with your friends, everyone agrees to give their favorite game controller to whomever reaches the highest level in the game first.
- Is this gambling?

- ANSWER: Yes
  - It is gambling because the winner receives something of value from the other players.

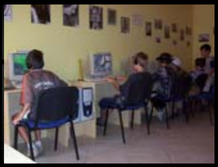

Is it gambling?

- Is it gambling to say to your friends, "I can make more free throws than you."?

- ANSWER: No.
- Competition is in most cases healthy. There are times, however, when competition can lead to risky gambling related behaviors.

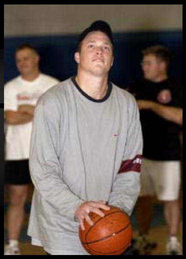

Gambling is...

Risking something of value, when the outcome is uncertain.

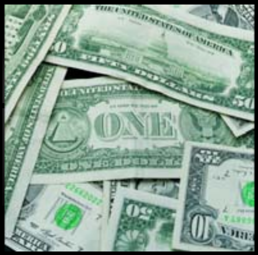

Misconceptions About Gambling

- True or False: It is easy to win back money you’ve lost gambling, you just have to gamble long enough for it to happen.

- ANSWER: False
- That is called “chasing the loss” and it only results in losing more money. Rarely do people win back the money they’ve lost gambling.

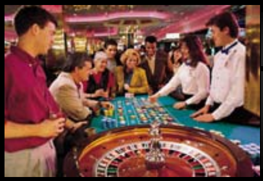

Misconceptions About Gambling

- If you spend $2 on a scratch card and you win another scratch card, how much money have you actually won?
  - a. You’ve lost $2
  - b. You’ve won $2
  - c. Nothing

- ANSWER: C = Nothing
- Even though you’ve won another card, you have not won any money. There is no guarantee the free card you’ve won will give you money.

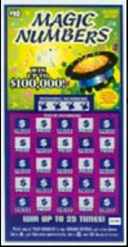

Are the Following Games of Skill or Games of Chance?

- Chess
- Baseball
- Bowling
- Basketball
- Pool
- Golf

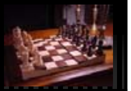

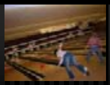

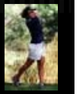

- ANSWER: GAMES OF SKILL
- These are all games that you can improve in, if you practice them.

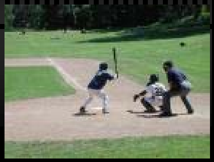

Are the Following Games of Skill or Games of Chance?

- Lotteries
- Bingo
- Roulette
- Keno
- Slot Machines
- Video Slot Machines
- Craps

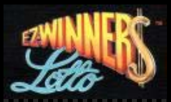

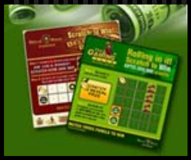

- ANSWER: GAMES OF CHANCE
- These games are all based on chance. Chance is uncontrollable. No one can do anything to improve their performance while playing these games.

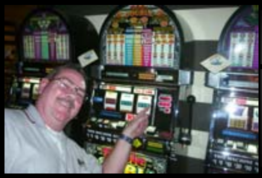

Games of Skill vs Games of Chance

- To be a smart gambler you should
  - a. Practice daily
  - b. Develop a strategy
  - c. Be careful, set limits and stick to them

- ANSWER: C = Be careful, set limits and stick to them.
- Gambling is based on chance. The only way to be a smart gambler, is to set betting limits and STOP when you've reached them.

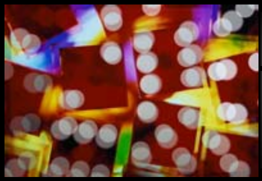

Games of Skill vs Games of Chance

- True or False: To increase your chance of winning while gambling, just follow your gut instincts.

- ANSWER: False
- Chance is uncontrollable. Your gut instincts cannot predict the outcome.

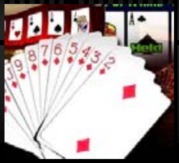

How Randomness Affects Gambling

- True or False: If you toss a coin 10 times and each time it comes up tails, there is a greater chance the next toss will be heads.

- ANSWER: False
- Tossing a coin is a random event. Both heads and tails have an equal chance of occurring on every toss, no matter what the outcome of previous tosses were.

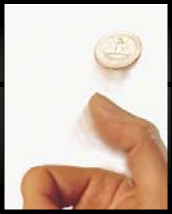

How Randomness Affects Gambling

- True or False: If you flip a coin long enough you can figure out the pattern and correctly guess what toss will come next.

- ANSWER: False
- Randomness is not the absence of patterns, it's the absence of <u>predictable</u> patterns. This is why you cannot correctly guess the next coin toss.

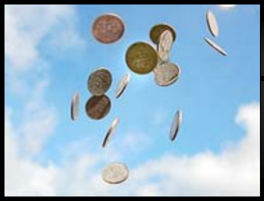

Rational vs Irrational Beliefs

- Is this a rational or irrational statement?
  - The more I tell my friends how much money I'm betting, the more I will impress them.

- ANSWER: Irrational statement
- True friends will not be impressed by gambling.

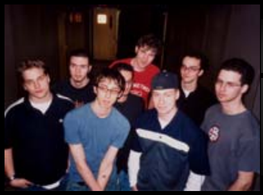

Rational vs Irrational Beliefs

- Rational or irrational?:
  - To become hooked on gambling, you have to be an adult who has spent many years at it.

- ANSWER: Irrational
- Adolescents can become problem gamblers very quickly.

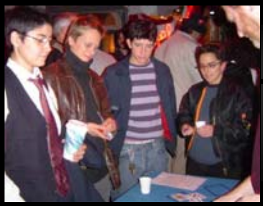

Rational vs Irrational Beliefs

- Gambling is ok if:
  - a. You have a perfect strategy to win
  - b. You need to make some fast money
  - c. You can afford to lose whatever you bet

- ANSWER: C = You can afford to lose whatever you bet.
- There is no strategy to win and you can't get rich quick by gambling.
- Plan how much you can afford to lose and then stop at that amount.

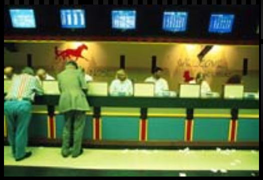

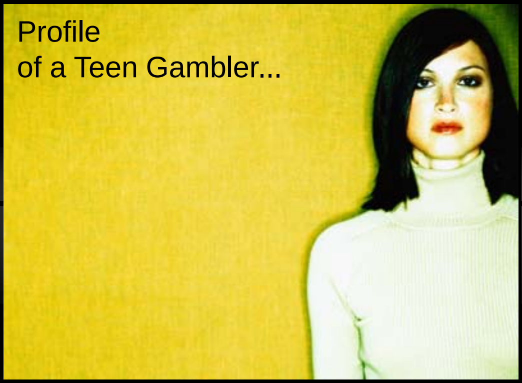

Profile of a Teen Gambler...

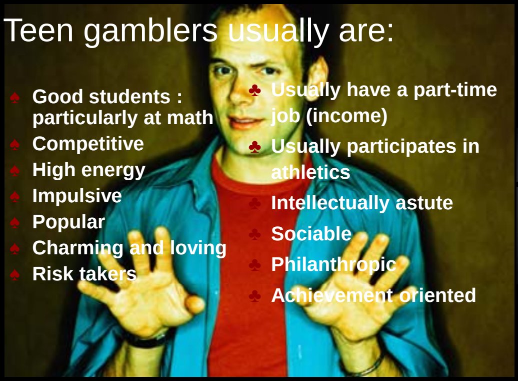

Teen gamblers usually are:

- Good students : particularly at math
- Competitive
- High energy
- Impulsive
- Popular
- Charming and loving
- Risk takers
- Usually have a part-time job (income)
- Usually participates in athletics
- Intellectually astute
- Sociable
- Philanthropic
- Achievement oriented

Problem Gambling – Signs & Symptoms

- Changes in behavior
- Changes in mood
- Unexplained need for money
- Carrying dice, cards, lottery tickets, etc.
- Unusual time spent watching sports on TV
- Gambling language in his/her vocabulary
- Late night phone calls from strangers
- Several calls to sports phone on telephone bill
- Having extra spending money

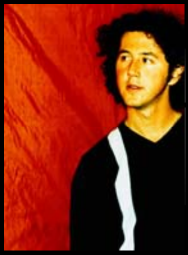

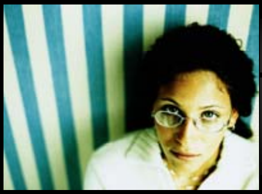

Progression

- Some wins
- Thinking about gambling when not gambling
- Needing to gamble more often and/or with more money
- Losing money and "chasing losses"
- Trying to stop gambling but not being able to do so
- Being irritable when not gambling
- Lying or committing crimes to support gambling
- Feeling desperate
- Losing everything, including hope

- Living life full of hope and enjoyment
- Thinking about school, family, work and the future
- Focusing on taking life one day at a time
- Being wiling to examine self and change things that need changing
- Developing productive coping skills and abilities
- Becoming honest with family and friends
- Actively seeking support and treatment
- Wanting life to be different

More information coming your way...

A CD ROM

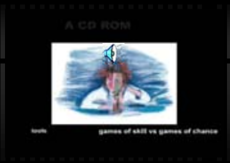

If You or Someone You Love...

has a problem with gambling or any other substance or behavior, there is help available 24 hours a day

The Illinois Institute for Addiction Recovery

1/800/522-3784

In Conclusion

- ✓ Post-test
