# Supplementary material for: Long-term effectiveness of a gambling intervention program among children in central Illinois
Source: PLoS One. 2019 Feb 11;14(2):e0212087. doi: 10.1371/journal.pone.0212087 (PMC6370280; doi:10.1371/journal.pone.0212087)
Supplement: S4 Appendix — (PDF) [file pone.0212087.s004.pdf]

Don't GAMBLE AWAY Our Future[SM]

Gambling Fact Sheets For Parents

Did you know…

Most teens with gambling problems are introduced to gambling by parents or other adults close to them?[1]

Most 9-14 year olds gamble in their homes with their families?[2]

Only 9% of parents surveyed were "very concerned" knowing that their children were gambling?[3]

Adolescents attempt to gamble at casinos regularly: In 1999, 53,400 young people were stopped at the doors, 39,000 youngsters were taken off the floor and into custody?[1]

Youth start by betting on[1]:

- Foul shots
- Running events
- Video games
- Card Games (usually poker and black jack)
- Dice games
- Football?

And Did You Know…

There is a downside to gambling?

Adolescent gambling begins between the ages 9-11?[1]

Gambling can be as harmful as drug use?

Gambling can result in self-destructive behavior including suicidal plans, substance abuse or dependence, and other high-risk behaviors?

Suicide attempt prevalence rates related to gambling can be as high as 15-24%?[4]

Teen Gamblers Typically Are[1]:

Competitive

Risk Takers

Achievement oriented

Dreaming of the "big win"

Able to rationalize/justify compulsive behavior

Focused on hobbies which include gambling

Insecure/low self-esteem

Charming and loving

Good students (particularly at math)

High energy

Employed with part-time jobs

Popular/Sociable

Involved in athletics

Organizers of activities

5409 N. KNOXVILLE, PEORIA, ILLINOIS 61614 • 309 691-1055 • 1-800-522 DRUG (3784) • www.addictionrecov.org • WE PUT TROUBLED LIVES BACK TOGETHER

Illinois Institute for Addiction Recovery
at Proctor Hospital with centers in Peoria, Bloomington, Springfield

This project was supported by Grant No. 2004-JL-FX-0162 awarded by the Office of Juvenile Justice and Delinquency Prevention, Office of Justice Programs, U.S. Department of Justice.
Points of view or opinions in this document are those of the author and do not necessarily represent the official position or policies of the U.S. Department of Justice.

Why Teens Gamble[1]?

Escape from other problems/reality
Peer pressure
Developmental factors
Winning = instant boost of confidence
Loneliness, depression, boredom
"Get rich quick"
Center of attention
Make friends by buying friends

Signs of Teen Gambling Include[1]:

Changes in behavior, moods, and availability of cash
Unexplained need for money
Carrying dice, cards, lottery tickets, etc.
Unusual time spent watching sports on TV
Gambling language in his/her vocabulary
Shifts in mood out of character
Late night phone calls from strangers
Several calls to sports phone on telephone bill
Unaccountable time away from home
Playing gambling games on the Internet
Having extra spending money
Weekly or daily card game in youngster's room
Boasting about winnings
Intense interest in gambling conversations
Marked decrease in activities that were once enjoyable
New interests in sporting events, newspapers, magazines and periodicals having to do with sporting events

Signs of Teen Gambling As the Problem Progresses[1]:

<u>At Home:</u>
Unexplained possessions
Missing cameras, TV's, jewelry
Money missing from bank accounts/wallets
Secrecy
Lies
New "unacceptable friends"

<u>At school:</u>
Tardiness
Truancy
Failing grades
Stealing

<u>In the community:</u>
Criminal activity/theft/robbery/assault
Vandalism
Gang activity
Threatening calls
Insurance fraud

If your son/daughter has these signs or symptoms, there could be a problem with gambling.

There is help available.

If you have questions or if you or someone you love may have a problem with gambling, please contact the Illinois Institute for Addiction Recovery at 1-800-522-3784 or go online at www.addictionrecov.org.

[1] Ed Looney, Executive Director Council on Compulsive Gambling of New Jersey, Inc.

[2] Ohlms, Terri Rodriques. (2000) "Teenage Gambling." *Paradigm Magazine*, Vol. 5 (2).

[3] Betty George, North American Training Institute. Minnesota Telephone survey on parental attitudes regarding their children gambling (age 15 or younger).

[4] Schneider, J. P. & Irons, R. R. "Treatment of gambling, Eating, and Sex Addictions." *http://www.prckansas.org/articles/gambling.htm*
