# Supplementary material for: Long-term effectiveness of a gambling intervention program among children in central Illinois
Source: PLoS One. 2019 Feb 11;14(2):e0212087. doi: 10.1371/journal.pone.0212087 (PMC6370280; doi:10.1371/journal.pone.0212087)
Supplement: S5 Appendix — (PDF) [file pone.0212087.s005.pdf]

Don't GAMBLE AWAY Our Future℠

5409 N. KNOXVILLE, PEORIA, ILLINOIS 61614 • 309 691-1055 • 1-800-522 DRUG (3784) • www.addictionrecov.org • WE PUT TROUBLED LIVES BACK TOGETHER

Dear Parent:

Did you know…

- The next generation is the first in modern American history to grow up in an era when gambling is legally sanctioned and culturally approved. Tragically, adolescents have proven extremely susceptible to the enticements of gambling?
  - Ronald Reno, Focus on the Family

- Youth are four times the risk of adults for developing pathological gambling?
  - Dr. Ken Winters, University of Minnesota

- An alarmingly high percentage of children and adolescents have reported engaging in gambling activities. We found 80.2% of adolescents between the ages of 12 and 17 reported having gambled (defined as wagering money) during the past 12 months, with 35.1% admitting gambling at least once a week. The data further revealed that while 55% of adolescents were casual or recreational gamblers, 13% reported having some gambling related problems and 4% to 6% had a serious problem?
  - Gupta & Derevesky, 1998

Today, children and adolescents are educated about the dangers inherent in tobacco, alcohol, and drug consumption. Few, however, are informed to understand the potentially addictive qualities inherent in gambling activities. The widely held belief that gambling is an innocuous behavior with few consequences has contributed to the lack of public awareness that gambling among children and adolescents can lead to a serious problem.

To make it worse, adolescents are at an increased risk of developing a gambling problem because of their stage of development, (e.g. vulnerability, developing ego), the appeal of risky/illegal behaviors, and having grown up in a society that regularly sends the message that gambling is an acceptable alternative to drugs and alcohol. Adolescents are attracted to gambling's promise to make them a "winner," a desire for high speed and instant gratification, alluring improved technologies, and a desire to "get away" with something. This vulnerability can quickly lead an adolescent into a painful gambling addiction before they or their loved ones recognize the loss of control.

Illinois Institute for Addiction Recovery
at Proctor Hospital with centers in Peoria, Bloomington, Springfield

This project was supported by Grant No. 2004-JL-FX-0162 awarded by the Office of Juvenile Justice and Delinquency Prevention, Office of Justice Programs, U.S. Department of Justice.
Points of view or opinions in this document are those of the author and do not necessarily represent the official position or policies of the U.S. Department of Justice.

Fortunately, there is evidence that prevention efforts can be used to curb pathological gambling behavior. The Illinois Institute for Addiction Recovery has developed a program to begin raising awareness and educating children & adolescents about the risks associated with gambling. We are showing a short presentation in your child's school discussing the definition of gambling, common gambling activities, and misconceptions about gambling. We introduce them to a profile of a pathological gambler including the progression associated with pathological gambling and how it affects students at home, school and in the community. We also identify where they can go for more information if needed. Your child also received an interactive CD-ROM that will continue to provide them with education about problem gambling in an engaging format.

All students participating in the presentation complete a pre- and post-test to determine the knowledge gained as a result of the prevention materials. Students (ages 12 years and older) are also assessed for their current gambling behavior through the Modified South Oaks Gambling Screen for Teens. All testing information is protected by state and federal laws regarding confidentiality and will only be used for data collection purposes.

Along with this letter, we've sent a packet of material related to problem gambling for your information. You can also access information on our interactive website at www.addictionrecov.org.

We are pleased to provide this important information to your student in their school. Thank you for your assistance in this endeavor.

Sincerely,

Phil Scherer, CAADC, PCGC, MISA II, BRI II
Director
Illinois Institute for Addiction Recovery
Proctor Hospital
