# Supplementary material for: Long-term effectiveness of a gambling intervention program among children in central Illinois
Source: PLoS One. 2019 Feb 11;14(2):e0212087. doi: 10.1371/journal.pone.0212087 (PMC6370280; doi:10.1371/journal.pone.0212087)
Supplement: S6 Appendix — (PDF) [file pone.0212087.s006.pdf]

Appendix 1

Don't Gamble Away Our Future
Pre-and Post-Test
High School

Name:____Age:____________

Grade:________________________________ Male / Female:________________

Please mark the following questions:

1. Which of the following are common gambling activities? **(MARK ALL ANSWERS)**
   a. Sports betting
   b. Bingo
   c. Lottery
   e. Video games
   f. All of the above

2. The definition of gambling is: betting money on something when the outcome is uncertain.
   a. True
   b. False

3. The following are games of chance: **(MARK ALL ANSWERS)**
   a. Chess
   b. Lottery
   c. Video slot machines
   d. Pool
   e. Both A and D
   f. Both B and C

4 When playing games of chance it helps to practice playing them daily, in order to improve.
   a. True
   b. False

5. If you flip a coin 5 times and you get heads 5 times in a row, you are most likely to get tails if you flip the coin again.
   a. True
   b. False
6. A random looking number (e.g., 12-5-23-7-19-34) is more likely to win than a number that has a sequence in it (e.g., 1-2-3-4-5-6).
   a. True
   b. False

7. When gambling in a casino, you can guarantee a win if you find out the probability of winning on the game you are playing.
    a. True
    b. False

8. When gambling in a casino, both you and the house have an equal chance of winning.
    a. True
    b. False

9. A gambling addiction is a lot less serious than a drug or alcohol addiction.
    a. True
    b. False

10. Which of the following statements are irrational beliefs regarding gambling: **(MARK ALL ANSWERS)**
    a. If I bet more money, I will win bigger amounts.
    b. People will be impressed if I tell them how much money I am winning while gambling.
    c. Only adults can become problem gamblers.
    d. The gamblers in Las Vegas who win use educated strategies.
    e. All of the above

11. Staying at the same slot machines improves your chances of winning.
    a. True
    b. False

12. Some signs & symptoms of a problem gambler are: **(MARK ALL ANSWERS)**
    a. Changes in mood and behavior
    b. Unexplained need for money
    c. Unusual time spent watching sports on TV
    d. Gambling language in his/her vocabulary
    e. All of the above

13. The progression of a problem gambler includes: **(MARK ALL ANSWERS)**
    a. Thinking about gambling when not gambling
    b. Trying to stop gambling, but being unable to do so
    c. Feeling desperate
    d. Losing everything, including hope
    e. All of the above

14. If someone you know has a gambling problem, you know where to go for help.
    a. True
    b. False
