# Supplementary material for: Long-term effectiveness of a gambling intervention program among children in central Illinois
Source: PLoS One. 2019 Feb 11;14(2):e0212087. doi: 10.1371/journal.pone.0212087 (PMC6370280; doi:10.1371/journal.pone.0212087)
Supplement: S7 Appendix — (PDF) [file pone.0212087.s007.pdf]

Appendix 2

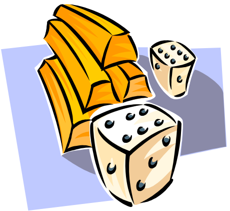

MODIFIED SOUTH OAKS GAMBLING SCREEN FOR TEENS (MSOGST)

1. Please indicate which of the following types of gambling you have done in your lifetime. For each type, mark one answer: "not at all," "less than once a week," or "once a week or more."

	not at all	less than once a week	once a week or more
a. Played cards for money	☐	☐	☐
b. Bet on horses, dogs, cockfights or other animals (at OTB, the track, with a bookie or with friends)	☐	☐	☐
c. Bet on sports (parlay cards, with a bookie, at Jai Alai or with friends)	☐	☐	☐
d. Played dice games (including craps, over and Under or other dice games) or dominos for money	☐	☐	☐
e. Gambled in a casino (legal or otherwise)	☐	☐	☐
f. Played the numbers or bet on lotteries	☐	☐	☐
g. Played bingo for money	☐	☐	☐
h. Played the stock and/or commodities market	☐	☐	☐
i. Played slot machines, poker machines or other gambling machines	☐	☐	☐
j. Bowled, shot pool, played golf or some other game of skill for money	☐	☐	☐
k. Pull-tabs or "paper" games other than lotteries	☐	☐	☐
l. Bet on Nintendo or other arcade games for money	☐	☐	☐
m. Some form of gambling not listed above	☐	☐	☐

2. What is the largest amount of money you have ever gambled with on any one day?

____never have gambled **____more than $100 up to $1,000**
____$1 or less **____more than $1.000 up to $10,000**
____more than $1 up to $10 **____more than $10,000**
____more than $10 up to $100 **____more than $10,000**

3. Check which of the following people in your life has (or had) a gambling problem.

____ father
____ mother
____ a brother or a sister
____ a grandparent
____ another relative
____ a friend or someone else important in my life
____ none of the above

4. When you gamble, how often do you go back another day to win back money you lost?

____ never
____ some of the time (less than half the time I lost)
____ most of the time I lost every time I lost

5. Have you ever claimed to be winning money gambling but weren't really? In fact, you lost?
____ never (or never gamble)
____ yes, less than half the time I lost
____ yes, most of the time

6. Do you feel you have ever had a problem with betting money or gambling?
____ no
____ yes, in the past but not now
____ yes

7. Did you ever gamble more than you intended to? ___yes ___no

8. Have people criticized your betting or told you that you had a gambling problem, regardless of whether you thought it was true or not? ___yes ___no

9. Have you ever felt guilty about the way you gamble or what happens when you gamble? ___yes ___no

10. Have you ever felt like you would like to stop betting money or gambling but didn't think you could? ___yes ___no

11. Have you ever hidden betting slips, lottery tickets, gambling money, IOU's or other signs of betting or gambling from your parents, close friends or other important people in your life? ___yes ___no

12. Have you argued with people you live with over how you handle money? ___yes ___no

13. (If you answered "yes" to question 12) Have money arguments ever centered on your gambling? ___yes ___no

14. Have you ever borrowed from someone and not paid them back as a result of your gambling? ___yes ___no

15. Have you ever lost time from school (or work) due to betting money or gambling? ___yes ___no

16. If you borrowed money to gamble or to pay gambling debts, who or where did you borrow from? (check "yes" or "no" for each):

a. from household money (lunch money).................	( ) Yes	( )No
b. from your parents..................................	( ) Yes	( )No
c. from other relatives...............................	( ) Yes	( )No
d. from banks, loan companies or credit unions.........	( ) Yes	( )No
e. from credit cards..................................	( ) Yes	( )No
f. from loan sharks ("Shylocks")......................	( ) Yes	( )No
g. you cashed in stocks, bonds or other securities.....	( ) Yes	( )No
h. you sold personal or family property...............	( ) Yes	( )No
i. you borrowed on your checking account (passed bad checks)................................	( ) Yes	( )No
j. you borrowed for gambling or betting but told someone it was for something else..................	( ) Yes	( )No

copyright, 2007 Illinois Institute for Addiction Recovery

SCORING

Scores on the SOGS itself are not determined by adding up the number of questions which show an "at risk" response:

Questions 1, 2, and 3 are not counted:

___ Question 4---most of the time I lose
or
every time I lose Q4=2

___ Question 5---yes, less than half the time I lose
or
yes, most of the time Q5=1, 2

___ Question 6---yes, in the past but not now
or
Yes Q6=1,2

___ Question 7---yes
___ Question 8---yes
___ Question 9---yes
___ Question 10---yes
___ Question 11---yes
___ Question 12---not counted
___ Question 13---yes
___ Question 14---yes
___ Question 15---yes
___ Question 14---yes
___ Question 15---yes
___ Question 16 a--yes
___ Question b---yes
___ Question c---yes
___ Question d---yes
___ Question e---yes
___ Question f---yes
___ Question g---yes
___ Question h---yes
___ Question i---yes
___ Question j--yes

Total=______

5 or more = probable pathological gambler

copyright, 1986 South Oaks Foundation
FORMS:SOAKS.YAU
